# The Effect of Shoulder and Knee Exercise Programmes on the Risk of Shoulder and Knee Injuries in Adolescent Elite Handball Players: A Three-Armed Cluster Randomised Controlled Trial

**DOI:** 10.1186/s40798-022-00478-z

**Published:** 2022-07-14

**Authors:** Martin Asker, Martin Hägglund, Markus Waldén, Henrik Källberg, Eva Skillgate

**Affiliations:** 1 Handball Research Group, Musculoskeletal & Sports Injury Epidemiology Center, Department of Health Promotion Science, Sophiahemmet University, Stockholm, Sweden; 2 Unit for Intervention and Implementation Research in Worker Health, Institute of Environmental Medicine, Karolinska Institutet, Solna, Sweden; 3 Naprapathögskolan, Scandinavian College of Naprapathic Manual Medicine, Stockholm, Sweden; 4 Sport Without Injury ProgrammE (SWIPE), Linköping University, Linköping, Sweden; 5 Unit of Physiotherapy, Department of Health, Medicine and Caring Sciences, Linköping University, Linköping, Sweden; 6 Unit of Community Medicine, Department of Health, Medicine and Caring Sciences, Linköping University, Linköping, Sweden; 7 GHP Ortho & Spine Center, Skåne, Malmö, Sweden; 8 Unit of Analysis, Department of Public Health, Analysis and Data Management, Public Health Agency of Sweden, Stockholm, Sweden

**Keywords:** Youth sport, Injury prevention, Sports medicine, Team sport, Neuromuscular training

## Abstract

**Background:**

The risk of injury in adolescent handball is high, and shoulder and knee injuries are among the most frequent and burdensome. The Swedish *Knee Control* programme reduced the risk of anterior cruciate ligament injuries in female youth football players and traumatic knee injuries in male and female youth floorball players. However, to date, *Knee Control* has not been evaluated in an elite youth sport setting. The literature on the prevention of shoulder injuries in sport is scarce, and there are to our knowledge no previous studies evaluating the preventative efficacy of injury prevention exercise programmes (IPEPs) on shoulder injuries in adolescent handball players.

**Objectives:**

To study the preventive efficacy of IPEPs on shoulder and knee injuries in adolescent elite handball players.

**Methods:**

Eighteen Swedish handball-profiled secondary schools (clusters) with players aged 15–19 years, 54% males were randomised into either the Shoulder Group or Knee Group (interventions) or a Control Group. Players in the Shoulder Group were instructed to perform the *Shoulder Control* programme, and  players in the Knee Group to perform the *Knee Control* programme, three times per week during May 2018 to May 2019. Control Group players continued their usual training. Outcomes were shoulder and knee injuries defined by the Oslo Sports Trauma Research Center Overuse Injury Questionnaire. Intention-to-treat analyses were performed using Cox regression models with hazard rate ratios (HRRs) with corresponding 95% confidence intervals (CI).

**Results:**

Six clusters (199 players) in the Shoulder Group, six clusters (216 players) in the Knee Group and six clusters (212 players) in the Control Group were included. There were 100 shoulder injuries and 156 knee injuries. The Shoulder Group had a 56% lower shoulder injury rate, HRR 0.44 (95% CI 0.29 to 0.68), and the Knee Group had a 31% lower knee injury rate, HRR 0.69 (95% CI 0.49 to 0.97) than the Control Group. The absolute risk reduction was 11% and 8%, and the number needed to treat was 9 and 13, respectively.

**Conclusions:**

Adolescent elite handball players who performed the *Shoulder Control* and the *Knee Control* programmes had a lower risk of shoulder and knee injuries, respectively, than players who continued their usual training. Further research on how these two programmes can be combined to reduce knee and shoulder injuries in a time effective way is warranted.

*Trial registration* ISRCTN15946352.

**Key points**

- The burden of knee and shoulder injuries in handball is high.
- The *Shoulder Control* programme reduces the risk and overall burden of shoulder injuries in adolescent elite handball players.
- The *Knee Control* programme reduces the risk and overall burden of knee injuries in adolescent elite handball players.

**Supplementary Information:**

The online version contains supplementary material available at 10.1186/s40798-022-00478-z.

## Background

The risk of injury in handball is high, and adolescent players have reported injury rates of 8.3 to 40.7 injuries per 1000 match hours and 0.6 to 3.7 injuries per 1,000 training hours [1–4]. Shoulder and knee injuries are among the most frequent and burdensome [1–6].

Injury prevention exercise programmes (IPEP) reduce the risk of sport injuries when used regularly, specifically acute knee ligament injures [7, 8]. The Swedish Knee Control programme (*Knee Control*) was developed in 2005 (Knäkontroll, SISU Idrottsböcker©, Sweden, 2005) and is a warm-up programme that focuses on lower limb and trunk strength, neuromuscular control, balance and jumping/landing technique. *Knee Control* contains six principal exercises: one-legged knee squat, pelvic lift, two-legged knee squat, the bench, the lunge and jumping/landing technique. Each exercise consists of four levels of difficulty with one additional partner exercise. *Knee Control* reduced the risk of anterior cruciate ligament (ACL) injuries by 64% in female youth football players and acute lower limb injuries by 45% in male and female youth floorball players [9, 10]. To date, *Knee Control* has not been evaluated in an elite youth sport setting. The literature on the prevention of upper limb sport injuries, and especially gradual onset (overuse) injuries, is scarce. A previous randomised controlled trial (RCT) on handball players showed a reduction of shoulder problems by 28% in male and female adult elite players, but no significant risk reduction when only including players without shoulder problems at baseline [11].

The objective of this cluster RCT was to investigate if a strengthening and throwing training programme for the shoulder (*Shoulder Control*), and a lower limb strength and neuromuscular control programme (*Knee Control*), reduce the rates of shoulder and knee injuries, respectively, in adolescent elite handball players. The hypotheses were that the *Shoulder Control* programme would reduce the shoulder injury rate, and that the *Knee Control* programme would reduce the knee injury rate.

## Methods

### Trial Design and Setting

This was a three-armed cluster RCT designed and reported in accordance with the CONSORT framework [12]. The trial was registered in the International Standard Randomised Controlled Trial Number (ISRCTN) registry in April 2018 prior to trial start (ISRCTN15946352), https://doi.org/10.1186/ISRCTN15946352. The trial was performed in a handball-profiled school setting, and the study population was followed for a calendar year (May 2018 to May 2019). A cluster design was chosen to minimise the risk of between-group contamination. A cluster was defined as all male and female players enrolled at the same handball-profiled school.

### Eligibility and Recruitment

Eligible for this trial were Swedish handball-profiled secondary schools with students aged 14 to 19 years that met the following three criteria: (1) a capacity for ≥ 30 handball-profiled students, (2) an even or near even enrolment of male and female players and (3) classified by the Swedish Handball Federation (SHF) as being at the highest elite level. After assessment for eligibility, the eligible schools were informed about the upcoming trial by the SHF by emails. The research leader (MA) then contacted the head coach of the schools over telephone to provide additional information and answer any questions.

Inclusion criteria at the player level were: (1) age 14 to 19 years and enrolled or accepted for enrolment at one of the eligible schools, (2) scored < 40 points at baseline on the Oslo Sports Trauma Research Center Overuse Injury Questionnaire (OSTRC-O) [13] regarding shoulder or knee problems in the preceding seven days (range 0–100 points) and (3) reported no shoulder or knee surgery in the preceding six months. Inclusion criteria 2 was to ensure that the participants did not have the primary outcome already at study start.

### Randomisation and Allocation of Concealment

Schools were randomised into one of three groups: Shoulder Group, Knee Group or Control Group. An independent research assistant performed the randomisation and prepared sealed envelopes with the sequence generation. The randomisation procedure was stratified by the number of players enrolled at each school: < 48 players (six schools), 48 to 66 players (nine schools) and > 66 players (three schools). Each school identification number was noted on an envelope that was divided into three strata specific piles. Three urns with three, six and nine folded cards, respectively, with the numbers 1, 2 or 3 indicating the arm, were prepared. To generate the random allocation sequence, one folded card at the time was taken from each stratum and put in the numbered envelope in each pile. The random allocation sequence was concealed from schools until all baseline data had been collected, and only the research leader knew the randomisation outcome. Due to the nature of the intervention, schools and players were not blinded to group allocation after the randomisation code was revealed.

### Baseline Data Collection

The research leader visited all included schools, explained the different steps of the trial and provided written information for the players (the same for all three groups).

At baseline, after receiving written informed consent to participate from the players (and legal guardians when needed) but before revealing the group allocation, players answered a questionnaire in paper format including questions on demographics, handball experience, participation in other sports, playing level, injury history and the OSTRC-O.

### Interventions

#### Shoulder Control

The *Shoulder Control* programme focuses on shoulder and trunk strength and control, trunk mobility and handball throwing load (velocity and frequency). It consists of five principal exercises that each has four levels of difficulty and an additional partner exercise. It also includes a throwing programme to be performed during the off-season and pre-season (June to August) consisting of four steps of progression in throwing velocity and number of throws. The players were instructed to start at level A and after one week progress to the next level, and so on until reaching the highest level–D (Additional file 1). Exercises in the *Shoulder Control* programme were based on previous studies on risk factors for shoulder injuries in handball players, where the most consistent risk factor is pre-season shoulder weakness [14–19] and were also inspired by previous exercise resources for handball (Knä- och Axelkontroll–Prestera bättre, SISU Idrottsböcker©, Sweden, 2007). Prior to trial start, a round table discussion with participants from the research group, handball coaches and experienced clinicians working in handball detailed the components and progressions of the programme. We aimed for exercises that were easy to perform on the handball court and easy to understand and progress for the coaches and players. The programme takes 10 to 15 min to perform and is meant to be used as a warm-up prior to handball training or match during the handball season. During the off-season, the programme is meant to serve as a strengthening programme. For more detailed information about *Shoulder Control*, see Additional file 1.

#### Knee Control

The *Knee Control* intervention was based largely on the original programme (Knäkontroll, SISU Idrottsböcker©, Sweden, 2005), but for this trial minor modifications were made to fit a handball setting (Additional file 2).

#### Intervention Implementation

The research leader held workshops for coaches and players at all intervention group schools at baseline (April 23 to May 31) and instructed the intervention programmes. Players and coaches were also provided with leaflets and videos describing all exercises and were instructed to start using the programmes directly after the workshops. The few players who could not attend the live workshop could review the recorded content online afterwards. Coaches were responsible for carrying out the intervention with their players during the school semesters. The players were responsible for carrying out the intervention as prescribed during the school breaks (on average seventeen calendar weeks).

Players were instructed to start the intervention programme at a difficulty level where they could perform the exercises with good quality, but still that the exercises were challenging. During the workshop, the players in the intervention group were instructed how to perform all exercises and the research leader helped the players identify an appropriate level where the exercise was performed with good quality, but still challenging. Players were instructed to progress to the next level when the exercise was not challenging anymore, e.g. if the push plus on the knees was effortless, they progressed to performing it on the toes. Intervention group players were instructed to perform the programme at least three times per week with three sets of 15–30 s per exercise during the off-season and pre-season (June to August 2018), and at least three times per week with two sets of 15–30 s per exercise during the handball season (September to May). The different length of the exercises (15–30 s) was suggested by coaches and clinicians and was used so that the players could use the same exercise regardless if it was used as a strengthening exercise during the off-season and pre-season period or as a warm-up exercise during the handball season. The players were instructed to perform the exercises with a pace of 1–2 s from the starting position of the exercise to the end position, e.g. for resisted external rotation in 90–90 position, 1 s concentric and 1 s eccentric, and trunk rotation in push up position, 2 s from starting position to end position (Additional file 1).

#### Control Group

The Control Group was instructed to train and play as usual and received no trial intervention. Coaches and players received information that if the programmes were efficacious, all schools were to be given instructions about both intervention programmes after the trial.

### Follow-Up

Follow-up data were collected from May 2018 to May 2019. Players were monitored during the follow-up period with weekly surveys including the OSTRC-O and additional questions on handball exposure (match and training hours), handball injuries and the amount of strength and conditioning training including both school and club trainings. Compliance to the programmes was measured via the weekly surveys by asking the players “How many times during the previous week have you completed the *Knee Control* programme” and “How many times during the previous week have you completed the *Shoulder Control* programme”, respectively. The surveys were administrated every Sunday at 6 p.m. in three ways: via an application (Briteback AB, version 2.0.7, Linköping, Sweden), via a short message service (SMS) and via email. Non-responders received a daily reminder via application notification, SMS and email for three days (Monday to Wednesday). On the Thursday, a research assistant phoned non-responders and took survey answers via telephone. When an injury with a sudden onset was reported, a clinician from the research group contacted the player via telephone to collect information about the injury using a standard injury report form (Additional file 3). The research assistants and clinicians were all blinded to group allocation. At the end of the trial in May 2019, the head coaches at the schools of the intervention groups reported how many organised handball training sessions per week they had performed during the trial period and how many times per week they performed the *Shoulder Control* or *Knee Control* programmes through an online survey.

### Injury Definitions and Outcome Measures

The outcomes were measured with the OSTRC-O [13]. The primary outcome “injury” was defined as reporting a score of 40 or more for the composite score of 0–100, as in other recent studies on handball players investigating risk factors for shoulder injuries [15, 19]. The composite score based on the OSTRC-O was chosen since it also captures pain. Another commonly used outcome based on the same questionnaire is “any shoulder problems” or “substantial shoulder problems” [6, 11, 13, 17], which were secondary outcomes in this study. The latter is based on the specific answers to questions about reductions in sports participation or performance only. All definitions and outcome measures are presented in Box 1

**Box 1:** Outcome measures and injury definitions

**Primary outcomes**
•* Shoulder and knee injury*—Reporting a score of ≥ 40 points for shoulder or knee problems with the OSTRC-O. The responses to each of the four questions in the OSTRC-O are given a value from 0 to 25, where 0 equals no problems and 25 equals the maximum level for each question •* Injury rate*—the number of first-time shoulder or knee injury events during the study follow-up per 1,000 h of handball play
**Secondary outcomes**
•* Time-loss shoulder and knee injury*—Reporting at least reduced participation or inability to participate due to a shoulder or knee problem with the OSTRC-O * Substantial shoulder and knee problems*—Reporting at least a moderate reduction in training volume or performance due to a shoulder or knee problem with the OSTRC-O •* Any shoulder and knee problem—*Reporting anything but “full participation without any shoulder/knee problem” with the OSTRC-O •* Weekly prevalence of substantial shoulder and knee problems*—The number of players who reported a substantial shoulder or knee problem with the OSTRC-O in each group divided by the total number of player reports in the group each week •* Weekly prevalence of any shoulder and knee problems*—The number of players who reported any shoulder or knee problem with the OSTRC-O in each group divided by the total number of player reports in the group each week
**Definitions**
•* Acute injury*—Injury with a sudden onset •* Gradual onset injury*—Injury not reported as having an acute onset

*OSTRC-O* Oslo Sports Trauma Research Center Overuse Injury Questionnaire

### Sample Size

We calculated the sample size based on the primary outcome and estimated that 18% of the players in the Control Group would report at least one shoulder injury and 25% at least one knee injury [20] during the season, and a 50% reduction in injury rates in each intervention group versus the Control Group [9, 10]. With adjustment for potential cluster effect, the required sample was a minimum of five clusters with on average 45 players in each of the three trial arms (total *n* = 675) to provide a minimum of 80% power (*α* = 0.05). Based on a previous study in this setting [20], we accounted for an estimated player drop-out rate of 5% and, therefore, we aimed to recruit at least 15 schools with approximately 710 players.

### Statistical Methods

Data were analysed according to the intention to treat principle. To compare the groups, Kaplan–Meier estimates were calculated, and hazard rate ratios (HRRs) with corresponding 95% confidence intervals (CI) were calculated for each outcome using Cox regression models (accounting for potential cluster effect). To evaluate the degree of within cluster dependence, we calculated the intraclass correlation coefficient (ICC) at baseline for cluster and training time using ANOVA adopted for ICC analysis. The significance level was set to *p* < 0.05. Time at risk was the number of hours of handball matches/training on the handball court between baseline and either the first shoulder or knee injury, respectively, or until censored or end of the study period. Players who quit the trial or reported a knee or shoulder injury, respectively, were censored for this specific injury outcome. Since time at risk was based on the number of hours exposed to handball, not on calendar time, players who reported other reasons for not fully participating in handball (e.g. school breaks, other school commitments, other injuries or illnesses) were not censored. No imputation of missing weekly reports was made. Absolute risk reduction (ARR) was calculated as the difference in cumulative injury risk for the whole follow-up time between the intervention groups and the Control Group. Number needed to treat (NNT) was calculated as the inverse of ARR. Weekly prevalence of any or substantial shoulder and knee problems was calculated as the number of players who reported any and substantial shoulder or knee problems, respectively, with the OSTRC-O in each group divided by the total number of player reports in the group each week. Number of weeks with shoulder and knee injury was calculated for each group. Nine players who did not respond to any weekly reports and therefore did not provide any exposure time or injury data were not included in the Cox regression analyses. All analyses were performed in R version 4.01 (R Core Team 2020,. R: A language and environment for statistical computing, R Foundation for Statistical Computing, Vienna, Austria) and the package “survival”. The statistician (HK) performing the analyses was blinded to group allocation.

### Patient and Public Involvement

The SHF was consulted in the planning and preparation of this study. Their involvement included inputs on the study plan and recruitment of participants. Handball coaches and experienced clinicians working in handball gave their inputs on the components and progression of the *Shoulder Control* programme. None of the above had any influence on the analyses, interpretation of the results or manuscript preparation.

## Results

**Fig. 1:**
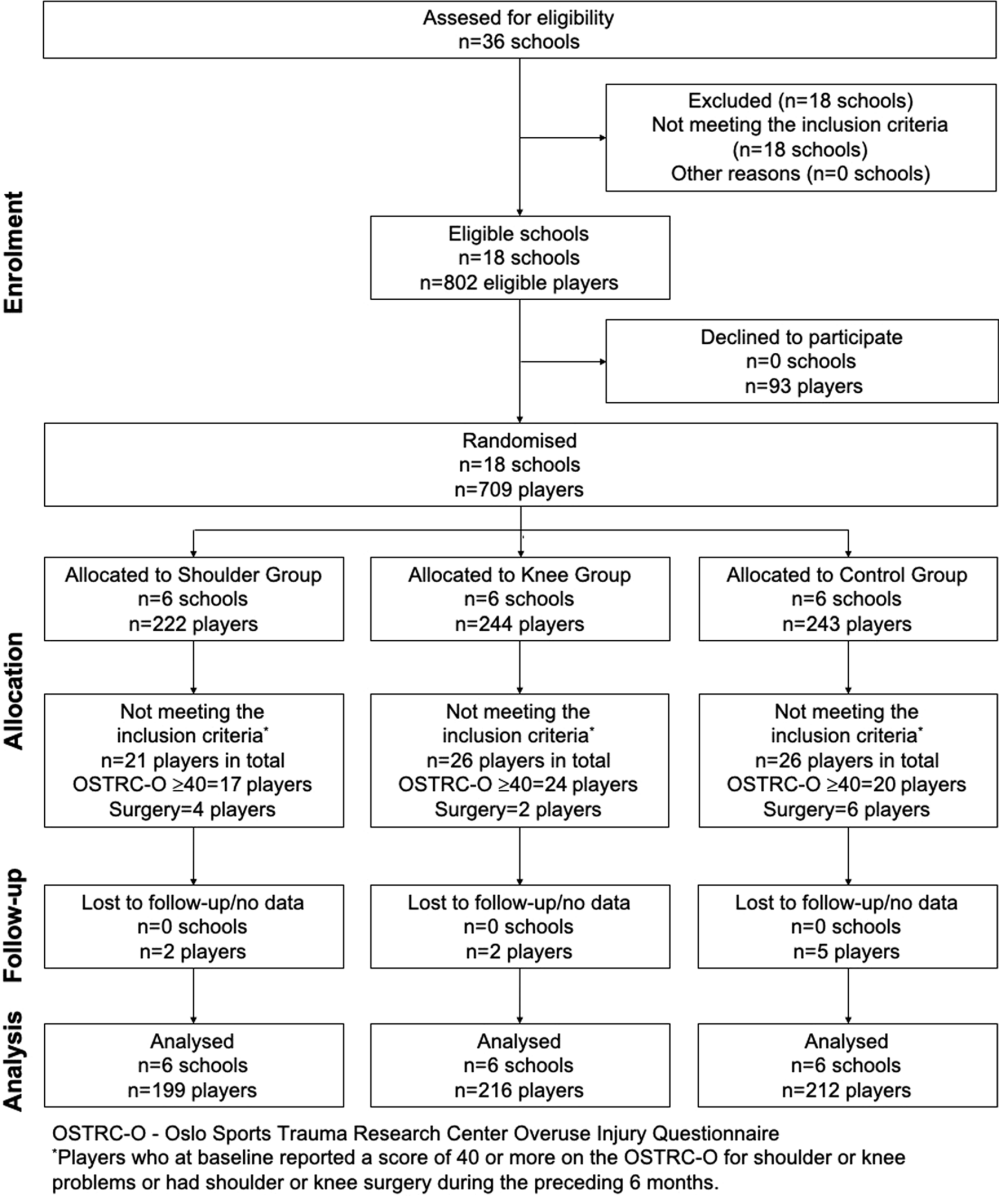
Flow chart describing the clusters (schools) and study population

Of the 36 handball-profiled schools in Sweden, 18 schools were eligible. All eligible schools (clusters) and 709 out of 802 eligible players (88%) from those 18 schools consented to participate in the trial and were randomised (Fig. 1). There were six schools in each group and no school dropped out during the trial, but 45 players from 16 schools dropped out: 14 players in the Shoulder Group (6%), 14 players in the Knee Group (6%) and 17 players in the Control Group (8%). The main reason in all groups was quitting the handball-profiled school.

Baseline characteristics of the players are presented in Table 1. A total of 24,517 weekly reports, including 11,194 handball match hours, 83,923 handball training hours and 88,015 strength and conditioning training hours, were registered with an average weekly response rate of 69% in the Shoulder Group, 71% in the Knee Group and 68% in the Control Group.

**Table 1:** Baseline characteristics

	Shoulder Group (*n* = 201)	Knee Group (*n* = 218)	Control Group (*n* = 217)
Age, years (SD)	16.5 (0.9)	16.5 (0.9)	16.5 (0.9)
Sex, number of male players, (%)	120 (60)	111 (51)	116 (53)
Mean handball experience, years (SD)	9.5 (2.0)	9.1 (2.3)	9.3 (2.1)
Performing specific neuromuscular training exercises for the knee on a regular basis previous season, *n* (%)	108 (54)	141 (65)	134 (62)
Performing specific shoulder strengthening exercises on a regular basis previous season, *n* (%)	82 (41)	71 (33)	77 (36)
Playing at national level, *n* (%)^*^	27 (13)	64 (29)	73 (34)
History of shoulder pain, *n* (%)^†^	97 (48)	103 (47)	112 (52)
History of knee pain, *n* (%)^†^	108 (54)	135 (62)	134 (62)
Playing position, n (%)			
Goalkeeper	24 (12)	21 (9)	27 (12)
Backcourt player	93 (46)	124 (57)	104 (48)
Wing player	51 (25)	45 (21)	51 (24)
Line player	33 (17)	28 (13)	35 (16)

*SD *standard deviation

^*^Players who had participated in a youth national team camp or tournament during the previous season

^†^Players who had experienced shoulder or knee pain respectively during handball at some point during the career

Handball match and training time and injuries during the follow-up period are presented in Additional file 4.

### Primary Outcomes

#### Shoulder Injury Rate

**Fig. 2:**
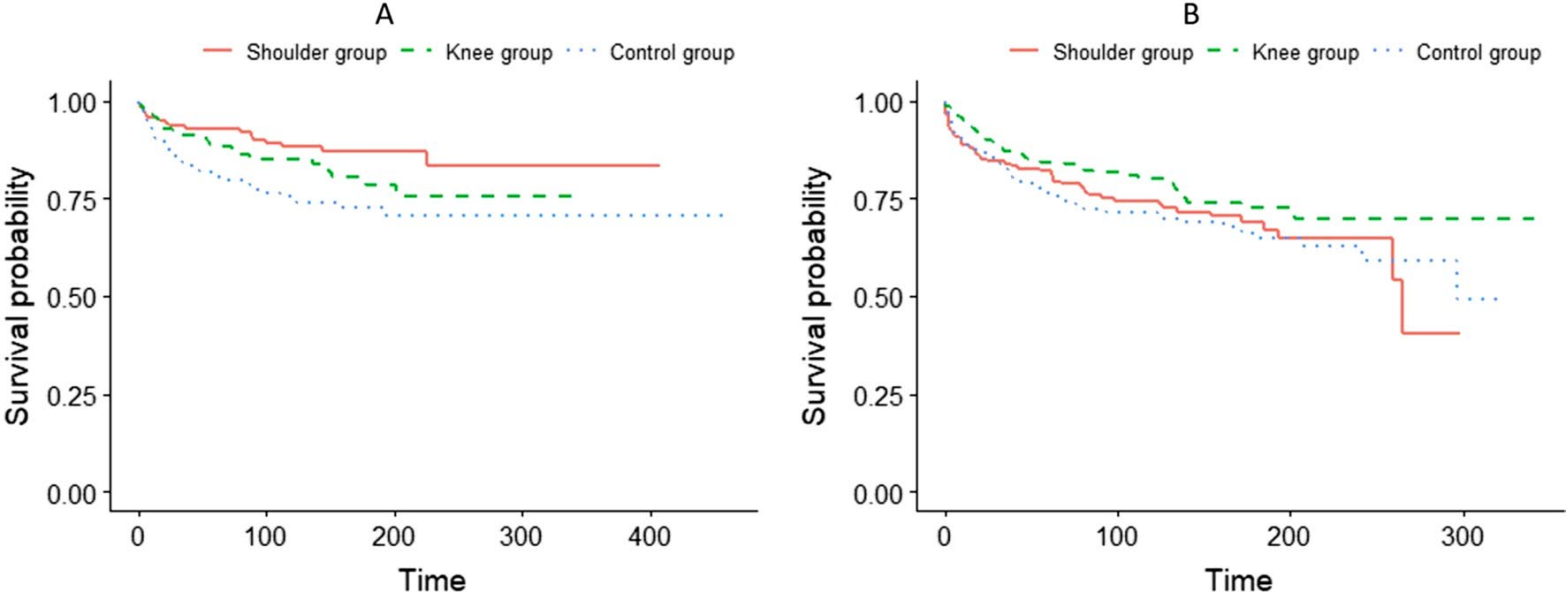
Kaplan–Meier estimates for shoulder injury (**A**) and knee injury (**B**). X-axis shows number of handball exposure hours

There were 100 new shoulder injuries, 21 in the Shoulder Group (82 weeks reported with injury), 33 in the Knee Group (198 weeks reported with injury) and 46 in the Control Group (191 weeks reported with injury). This corresponded to an injury rate of 0.8 (95% CI 0.5 to 1.2) per 1,000 handball hours in the Shoulder Group, 1.3 (95% CI 0.9 to 1.8) in the Knee Group and 1.8 (95% CI 1.3 to 2.4) in the Control Group. The Shoulder Group had a 56% lower shoulder injury rate than the Control Group, HRR 0.44 (95% CI 0.29 to 0.68). Kaplan–Meier estimates for shoulder injuries are presented in Fig. 2. The ARR between the Shoulder Group and Control Group was 11% (95% CI 4 to 18), and the NNT was 9 (95% CI 24 to 6). The relative rates of shoulder injuries between the three groups are presented in Table 2.

**Table 2:** Hazard rate ratios for shoulder and knee injuries/problems between the three trial arms

	HRR	95% CI	P-value	
Primary outcomes				
Shoulder injury^*^				
Shoulder Group versus Control Group	0.44	0.29 to 0.68	< 0.001	
Shoulder Group versus Knee Group	0.66	0.38 to 1.14	0.14	
Knee Group versus Control Group	0.66	0.38 to 1.13	0.13	
Knee injury^*^				
Knee Group vs Control Group	0.69	0.49 to 0.97	0.03	
Knee Group versus Shoulder Group	0.74	0.54 to 1.01	0.06	
Shoulder Group versus Control Group	0.94	0.69 to 1.29	0.71	
Secondary outcomes				
*Time-loss shoulder injury*^†^				
Shoulder Group versus Control Group	0.44	0.30 to 0.65	< 0.001	
Shoulder Group versus Knee Group	0.60	0.38 to 0.94	0.03	
Knee Group versus Control Group	0.74	0.53 to 1.03	0.07	
*Substantial shoulder problem* ^‡^				
Shoulder Group versus Control Group	0.51	0.30 to 0.87	0.01	
Shoulder Group versus Knee Group	0.63	0.38 to 1.07	0.09	
Knee Group versus Control Group	0.80	0.50 to 1.29	0.36	
*Any shoulder problem*^§^				
Shoulder Group versus Control Group	0.49	0.29 to 0.83	0.01	
Shoulder Group versus Knee Group	0.65	0.42 to 0.99	<0.05	
Knee Group versus Control Group	0.80	0.51 to 1.11	0.16	
*Time-loss knee injury*^†^				
Knee Group versus Control Group	0.62	0.46 to 0.83	0.002	
Knee Group versus Shoulder Group	0.76	0.59 to 0.99	0.04	
Shoulder Group versus Control Group	0.82	0.64 to 1.05	0.12	
*Substantial knee problem*^‡^				
Knee Group versus Control Group	0.61	0.44 to 0.85	0.003	
Knee Group versus Shoulder Group	0.68	0.56 to 0.83	< 0.001	
Shoulder Group versus Control Group	0.90	0.65 to 1.25	0.53	
*Any knee problem*^§^				
Knee Group versus Control Group	0.77	0.54 to 1.12	0.17	
Knee Group versus Shoulder Group	1.43	1.15 to 1.79	0.002	
Shoulder Group versus Control Group	0.54	0.34 to 0.72	< 0.001	

*CI* confidence interval, *HRR *hazard rate ratio

^*^Reporting a score of 40 points or more for shoulder or knee problems, respectively with the Oslo Sports Trauma Research Center Overuse Injury Questionnaire (OSTRC-O)

^†^Reporting at least reduced participation or inability to participate due to a shoulder or knee problems, respectively with the OSTRC-O

^‡^Reporting at least a moderate reduction in training volume or performance due to a shoulder or knee problems, respectively with the OSTRC-O

^§^Reporting anything but “full participation without any shoulder or knee problems”, respectively with the OSTRC-O

#### Knee Injury Rate

There were 156 new knee injuries, 44 in the Knee Group (202 weeks with injury), 53 in the Shoulder Group (337 weeks with injury) and 59 in the Control Group (380 weeks with injury). This corresponded to an injury rate of 1.7 (95% CI 1.3 to 2.3) per 1,000 handball hours in the Knee Group, 2.3 (95% CI 1.7 to 3.0) in the Shoulder Group and 2.5 (95% CI 1.9 to 3.2) in the Control Group. The Knee Group had a 31% lower knee injury rate than the Control Group, HRR 0.69 (95% CI 0.49 to 0.97). Kaplan–Meier estimates for knee injuries are presented in Fig. 2. The ARR between the Knee Group and Control Group was 8% (95% CI -1 to 16), and the NNT was 13 (95% CI -162 to 6). The relative rates of knee injuries between the three groups are presented in Table 2.

### Secondary outcomes

#### Shoulder and Knee Time-Loss Injuries

Compared with the Control Group, players in the Shoulder Group had a 56% lower rate of time-loss shoulder injury, a 49% lower rate of substantial shoulder problems and a 51% lower rate of any shoulder problems (Table 2). Compared with the Control Group, the Knee Group had a 38% lower rate of time-loss knee injury, a 39% lower rate of substantial knee problems and a 23% lower rate of any knee problems (Table 2).

#### Prevalence of Shoulder and Knee Problems

The average weekly prevalence of any shoulder problem and substantial shoulder problems during the trial period (May to May) was 3% (95% CI 1 to 7) and 1% (95% CI 0 to 4) in the Shoulder Group, 6% (95% CI 3 to 10) and 2% (95% CI 1 to 5) in the Knee Group and 8% (95% CI 5 to 13) and 2% (95% CI 1 to 5) in the Control Group, respectively.

The average weekly prevalence of any knee problem and substantial knee problems during the trial period (May to May) was 6% (95% CI 3 to 10) and 2% (95% CI 1 to 5) in the Knee Group, 9% (95% CI 5 to 14) and 4% (95% CI 2 to 8) in the Shoulder Group and 11% (95% CI 7 to 16) and 4% (95% CI 2 to 8) in the Control Group.

The weekly prevalence of any and substantial shoulder and knee problems in the three groups during the trial period (May to May) is presented week by week in Additional file 5.

### Exposure and Compliance

Players in the Shoulder Group completed *Shoulder Control* (including the throwing programme June to August) in mean 1.3 (± 1.3) times per week and players in the Knee Group completed *Knee Control* in mean 1.6 (± 1.4) times per week. The schools in the Shoulder Group performed *Shoulder Control* on average 1.8 (± 0.8) times per week and schools in the Knee Group performed *Knee Control* on average 2.2 (± 0.8) times per week during the handball season (September to May). Schools had on average 2.2 (± 0.4) handball training sessions per week during the handball season (September to May).

## Discussion

The principal finding of this trial on adolescent elite handball players was that the IPEPs *Shoulder Control* and *Knee Control* reduced the risk of shoulder and knee injuries, respectively. Moreover, the Shoulder Group reported less than half the average weekly prevalence of shoulder problems and less total weeks of shoulder injuries compared with the Control Group during the trial period. Further the Knee Group reported almost half the average weekly prevalence of knee problems and less total weeks of knee injury compared with the Control Group during the trial period. This indicates that the programmes not only reduce the rate of new injuries, but also reduce the overall shoulder and knee injury burden.

Interestingly, even though many players reported using shoulder training programmes and knee warm-up programmes in the previous season, and that during the season they reported frequent strength and conditioning work, the implementation of specific shoulder and knee training interventions still led to a substantially reduced risk of shoulder and knee injuries.

### Shoulder Injuries

*Shoulder Control* was efficacious in reducing shoulder injuries with less than half the rate compared with the Control Group. Players in the Knee Group, however, had no reduced rate of shoulder injuries, which suggests that shoulder-targeted exercises are needed to prevent shoulder injuries. *Shoulder Control* also reduced the prevalence of shoulder problems which is in line with a previous study in senior elite players in Norway where a shoulder IPEP reduced the prevalence of shoulder problems [11]. The weekly prevalence of shoulder problems reported in this trial is lower compared to what has been reported in our previous study on a similar population [6]. However, in the present trial the weekly average prevalence is based on a whole calendar year in contrast to our previous study, which reported weekly prevalence during the competitive season only. Moreover, the average weekly response rate in previous study was higher (93%) compared to the present study, which could have affected the average weekly prevalence.

### Knee Injuries

The Knee Group had 31% lower rate of knee injury than the Control Group, which is in line with previous studies in handball and other team sports [2, 9, 10, 21]. The effect of *Knee Control* on knee injuries is thus somewhat lower than expected based on a previous study of *Knee Control* in similar-aged female football players in Sweden and a recent meta-analysis [8, 9], and there can be several explanations for this. First, in contrast to most previous studies, we included both acute and gradual onset injuries and most of the injuries were gradual onset injuries which could explain the lower effect [10]. Second, many players reported already using neuromuscular training exercises for the knee at baseline. Third, our trial included not only the handball season but also the pre-season where handball players normally perform heavy lower extremity exercises, which could increase the overall incidence of knee problems by an increased workload. As indicated by the weekly prevalence of knee problems (Additional file 5), it is evident that the relative risk would have been lower if the study period had included only the handball season (September to May).

*Shoulder Control* had no effect on the risk of knee injuries which indicates that to prevent knee injuries the exercises should target the lower limb and trunk. As with shoulder injuries, the weekly prevalence of substantial knee problems was quite stable with a reduction in prevalence in the end of the season compared to the Control Group. These findings expand on a previous study on youth floorball players in Sweden that also showed the same steady reduction in weekly prevalence [10].

### Performance and Compliance of *Shoulder Control *and *Knee Control*

On average, players performed the training programmes half of the recommended number of sessions (1.3 and 1.6 sessions/week), likely because they only had on average 2.2 training sessions per week during school hours. The intervention dose is similar to previous prevention RCTs in team ball setting (1.0 to1.6 sessions/week) [10, 11, 22]. Despite not reaching the instructed number of sessions per week, both interventions showed significant effects.

Time is one of the most important barriers for completing the IPEPs [23], and performing both *Knee Control* and *Shoulder Control* would take approximately 20 min in addition to the regular handball warm-up. However, some of the exercises in Knee Control and Shoulder Control, e.g. trunk strength exercises, are quite similar and future studies should investigate if these programmes can be combined and limit the total number of exercises and still be effective in reducing shoulder and knee injury risk.

### Strengths and Limitations

There are several strengths of this trial. First, the study design is the gold standard for our main research question. We performed a cluster randomisation based on school instead of individual player randomisation which minimised the risk of contamination and a potential misclassification of the intervention exposure. Second, all eligible schools accepted to participate, and 88% of players in the schools participated. This indicates that our trial has high external validity with a representative sample of the population of healthy adolescent elite handball players. Also, together with a low and even attrition rate in the three arms, this speaks for a low risk of selection bias. Third, by using a valid measurement tool the risk of misclassification of the outcome is low. Fourth, with the three-armed design we were able to compare two interventions both with a Control Group and with each other. By doing so, we could evaluate the efficacy of the interventions not primarily constructed to target the specific injury, i.e. the efficacy of *Shoulder Control* on knee injuries and the efficacy of *Knee Control* on shoulder injuries.

This trial is not without limitations. First, since most injuries were self-reported, this trial cannot evaluate the effect related to any specific shoulder and knee injury diagnoses. Moreover, the possibility to classify if the injury reported with the OSTRC-O was the same injury or a new or a recurrent injury is limited, especially in players who did not respond to all weekly reports. Therefore, to account for multiple injury events during the season we also presented injury prevalence data and total weeks with reported shoulder and knee injury in the three groups to compliment the Cox regression analyses. Moreover, the OSTRC-O composite severity score is created based on the answers to the four different questions, which has been criticised recently [24]. Some studies have primarily used substantial problems/injuries instead [6, 10, 11, 17, 25], which was used as a secondary outcome in our study. Importantly, consistently for primary and secondary outcomes, *Shoulder Control* reduced the risk of shoulder injuries and *Knee Control* reduced the risk of knee injuries. The association for any knee problems was not statistically significant. In previous studies no risk reduction was seen when “any problems” were used as outcome [10]. One explanation for this could be that “any problems” also include knee stiffness or ache, which is not uncommon to experience when starting *Knee control* and therefore the time to first reported any knee problem is potentially shorter in the Knee Group. Further, the overall burden of any knee problems over the trial period was markedly lower in the Knee Group as indicated by the number of weeks with knee injury and weekly prevalence of any knee problems (Additional file 5). Second, this trial was underpowered for subgroup analyses (e.g. sex or injury onset) and coupled to that we had more clusters than planned (18 vs. 15), but somewhat fewer players (627 vs. 675) compared with our a priori sample size estimation. Third, due to the nature of the interventions, the schools and players were not blinded to group allocation. Therefore, the expectations on intervention outcomes could differ between the three arms, but this risk is probably low in a prevention RCT. In our trial, we had fair number of relatively small clusters (18 clusters with on average 35 participants) and the cluster effect was very small and were accounted for in the analyses, and hence, we judge that our main findings are not explained by the cluster design of the trial. Finally, there were some group differences in baseline characteristics, constituting a risk of confounding despite the study design. The Shoulder Group had a higher percentage of male players compared to the Control Group. Previous studies have shown conflicting results on sex difference in the occurrence of shoulder injuries in handball players. [3, 6, 11, 18]. However, in this study there were no difference in the cumulative incidence of shoulder injuries between male and female players (Additional file 4). Another baseline difference was that the Shoulder Group had a lower proportion of players at a national level compared to the Control Group. Previous studies have reported no difference in the occurrence of shoulder problems between playing levels in adolescent and professional handball [6, 15]. The Knee Group had a higher percentage of backcourt players compared to the Control Group. There are to our knowledge no studies reporting the prevalence or incidence of knee injuries based on playing position in handball. However, based on the demands on backcourt player one could assume that the risk of knee injury would be higher in backcourt players compared to other playing positions in handball, as shown for shoulder injuries [6]. However, if this is true, the HRR for the Knee Group would probably be even lower with a more even distribution of backcourt players between the Knee Group and the Control Group. Altogether, this indicates that confounding is not an important threat to the trial validity.

## Conclusion

Adolescent elite handball players who performed the *Shoulder Control* programme and the *Knee Control* programme had a lower risk of shoulder and knee injuries, respectively, compared with players who continued their usual training. Further research on how these two programmes can be combined to reduce knee and shoulder injuries in a time effective way is warranted.

## Supplementary Information

**Additional file 1:** *Shoulder Control* exercise programme.

**Additional file 2:** *Knee Control* exercise programme.

**Additional file 3:** Acute injury report card.

**Additional file 4:** Handball match and training time and number of shoulder and knee injuries and problems during the follow-up.

**Additional file 5:** Weekly prevalence of shoulder and knee problems.

## Data Availability

The datasets generated during and/or analysed during the current study are available from the corresponding author on reasonable request.
