# Supplementary material for: The Effect of Shoulder and Knee Exercise Programmes on the Risk of Shoulder and Knee Injuries in Adolescent Elite Handball Players: A Three-Armed Cluster Randomised Controlled Trial
Source: Sports Med Open. 2022 Jul 14;8:91. doi: 10.1186/s40798-022-00478-z (PMC9283550; doi:10.1186/s40798-022-00478-z)
Supplement: Supplementary file 1 — Additional file 1: Shoulder Control exercise programme. [file 40798_2022_478_MOESM1_ESM.pdf]

Supplementary file 1. Shoulder Control exercise programme

The effect of shoulder and knee exercise programmes on the risk of shoulder and knee injuries in adolescent elite handball players: a three-armed cluster randomised controlled trial.

Martin Asker [1,2,3], Martin Hägglund [4,5], Markus Waldén [4,6,7], Henrik Källberg [1,8], Eva Skillgate [1,2]

[1] Handball Research Group, Musculoskeletal & Sports Injury Epidemiology Center, Department of health promotion science, Sophiahemmet University, Stockholm, Sweden

[2] Unit for Intervention and Implementation Research in worker health, Institute of Environmental Medicine, Karolinska Institutet, Solna, Sweden

[3] Naprapathögskolan, Scandinavian College of Naprapathic Manual Medicine, Stockholm, Sweden

[4] Sport Without Injury ProgrammE (SWIPE), Linköping University, Linköping, Sweden

[5] Unit of Physiotherapy, Department of Health, Medicine and Caring Sciences, Linköping University, Linköping, Sweden

[6] Unit of Community Medicine, Department of Health, Medicine and Caring Sciences, Linköping University, Linköping, Sweden

[7] GHP Ortho & Spine Center Skåne, Malmö, Sweden

[8] Unit of analysis, Department of Public Health, Analysis and Data Management, Public Health Agency of Sweden, Stockholm, Sweden

Corresponding author:
Martin Asker
martin.asker@shh.se

Supplementary file 1. Shoulder Control exercise programme

This programme is inspired by previous exercise resources for handball (Knä- och Axelkontroll – Prestera bättre SISU Idrottsböcker, Sweden 2007) and the aim of the programme is to increase strength, control and range of motion of the shoulder and upper body.

Choose one exercise from each category 1-5 at a difficulty level where you can perform the exercise with good technique but it is still challenging. During the off-season and pre-season (June to August) the programme is performed 3 times per week with 3 sets of each exercise, with a pace of 1-2 second from starting position to end position. During the off-season you also perform the throwing programme (exercise 6).

During the handball season (September to May) the programme is meant to be performed in conjunction with the normal handball warm-up. During the handball season the programme is performed in 2 sets of each exercise, with a pace of 1-2 second from starting position to end-position.

1. Shoulder strength/control – Part 1

Level A — **Y-raises with an elastic band** — **2-3x30 seconds**

Instructions: *Stand shoulder wide with an elastic band fixated in front on ground level. With slightly bent elbows, lift the arms so they shape a "Y" and go back to the starting position*

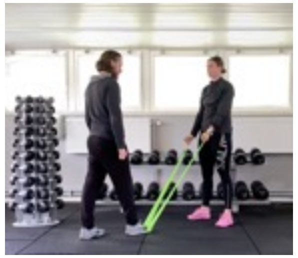
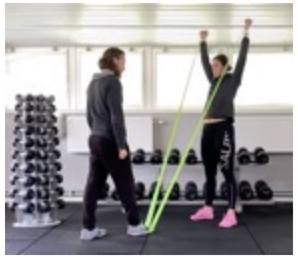

Level B — **Archer draws with an elastic band** — **2-3x30 seconds**

Instructions: *Stand with an elastic band fixated in front of the body. Pull the band and rotate the upper body "draw the bow" and go back to the starting position*

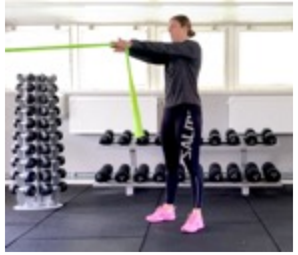
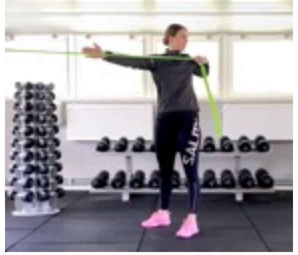

Level C — **Resisted external rotation in 90-90 position** — **2-3x30 seconds**

Instructions: *Stand shoulder wide with an elastic band fixated in front of the body. Put the arm in a "90-90 position" with the forearm pointing forward. Pull the band and rotate arm backward until the forearm pointing upward and go back*

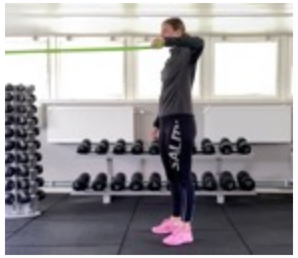
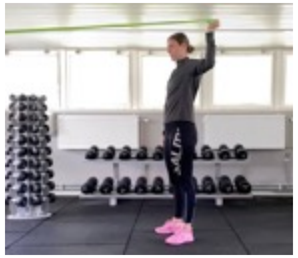

Level D — **Eccentric diagonal patterns – "Lower the arm"** — **2-3x30 seconds**

Instructions: *Stand with one foot in front of the other with an elastic band fixated in front on ground level. With help form the other arm, put the the arm in a throwing position. Slowly lower the arm*

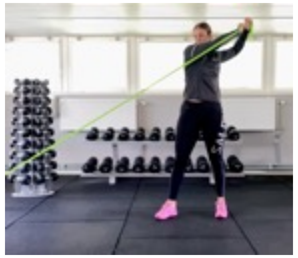
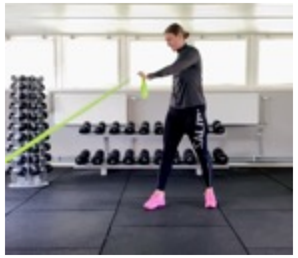

Partner exercise — **Reversed arm wrestling – "Clock to clock"** — **2-3x15 seconds**

Instructions: *Stand shoulder wide beside the partner. Put the arm in a throwing position and push the wrist against the partner´s wrist.*

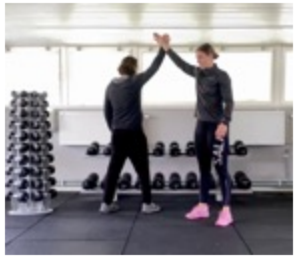

2. Shoulder strength/control - Part 2

Level A — **Push plus on knees and forearms** — **2-3x30 seconds**

Instructions: *Elbows are placed beneath the shoulder. Push the upper body up, protract the shoulder and hold for a second and go back to the starting position*

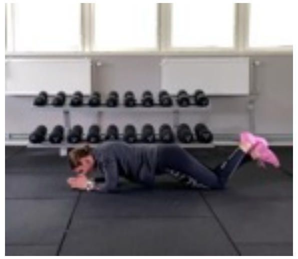
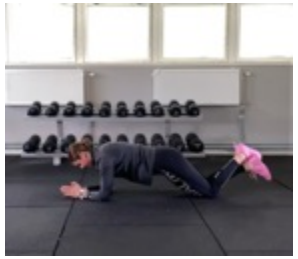

Level B — **Overhead elastic band pull apart** — **2-3x30 seconds**

Instructions: *Stand shoulder wide with the arms in an overhead position. Pull the band apart and hold for a second and then go back to the starting position*

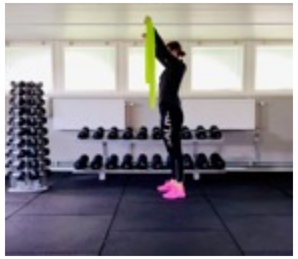
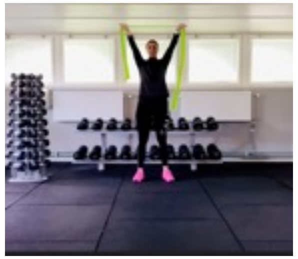

Level C — **Bench to push up** — **2-3x30 seconds**

Instructions: *Elbows are placed beneath the shoulder. Push the upper body up, protract the shoulder and shift the position to a push up position and back to the starting position again.*

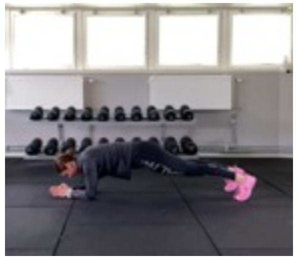
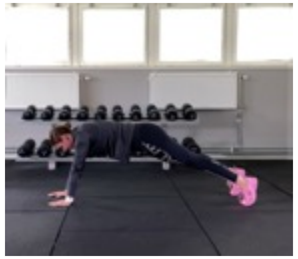

Level D — **Superman walkouts** — **2-3x15-30 seconds**

Instructions: *Stand in a push up position. Move the feet as far as possible while maintaining the body in a straight line*

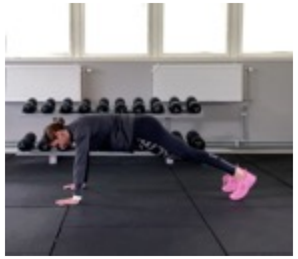
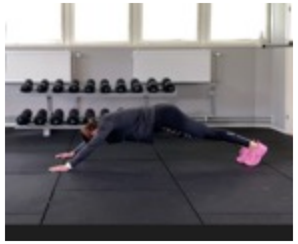

Partner exercise — **Wheelbarrow with push plus/bench to push up** — **2-3x15-30 seconds**

Instructions: *Same as level C but the partner support the ankles*

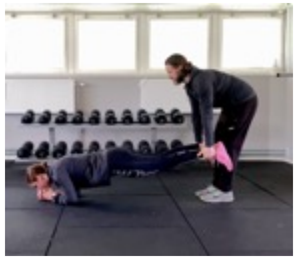
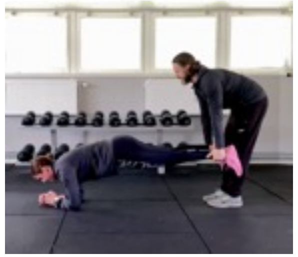

3. Upper body mobility

Level A — **Supine rotations** — **2-3x30 seconds**

Instructions: *Lay in a supine position with knees bent and feet on the ground. Put the hands together in front of the chest. Rotate the upper body as far as possible while maintaining the hands together.*

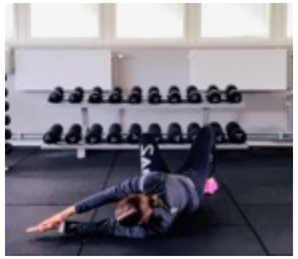
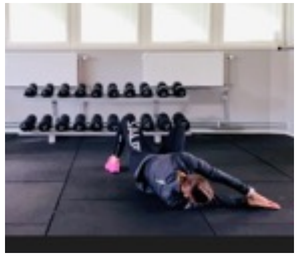

Level B — **Rotations in a bench position on knees** — **2-3x30 seconds**

Instructions: *Elbows are placed beneath the shoulder and knees on the ground. Rotate the upper body by putting one arm under the body as far as possible and then rotate to the opposite direction.*

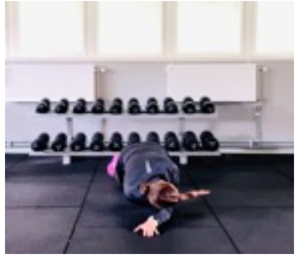
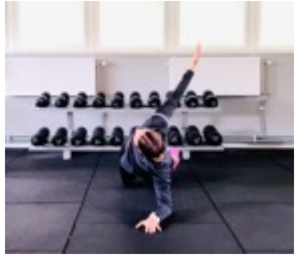

Level C — **Rotation in a bench position on toes** — **2-3x15-30 seconds**

Instructions: *Same as Level B but support on toes instead of knees*

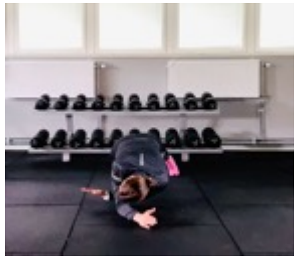
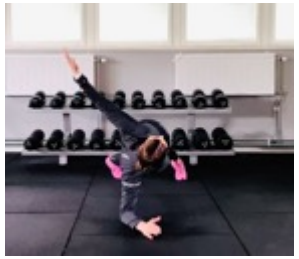

Level D — **Rotations in a push up position** — **2-3x15-30 seconds**

Instructions: *Same as Level B but in a push up position instead*

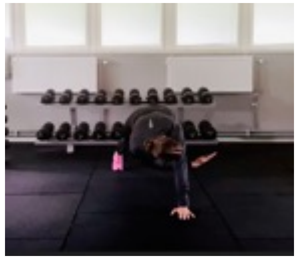
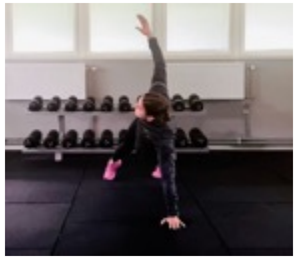

Partner exercise — **Rotation in push up position with passes** — **2-3x15-30 seconds**

Instructions: *Same as Level D but in the end position pass the ball to the partner*

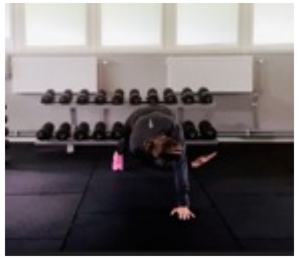
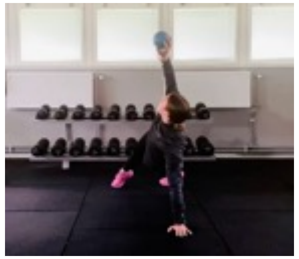

4. The Diver with one arm in overhead positions

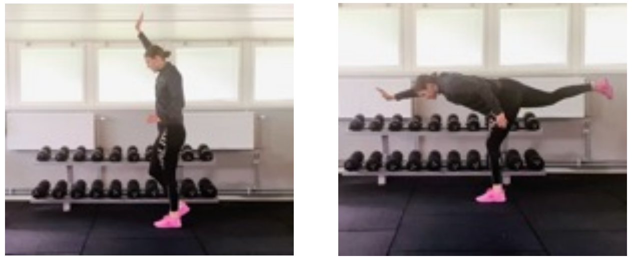

Level A **One arm diver in overhead position** **2-3x30 seconds**

Instructions: *Stand on one foot with the arm in an overhead position. Bend forward while maintaining the body in a straight line, performing a "T" and then go back to the starting position.*

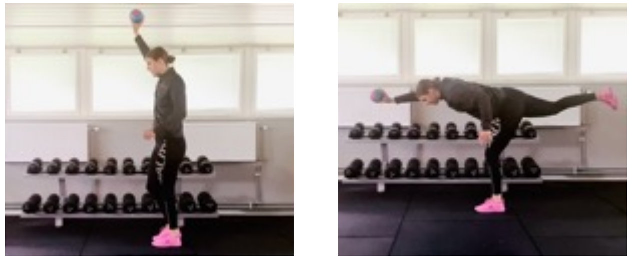

Level B **One arm diver in overhead position with a ball** **2-3x30 seconds**

Instructions: *Same as level A but holding a ball*

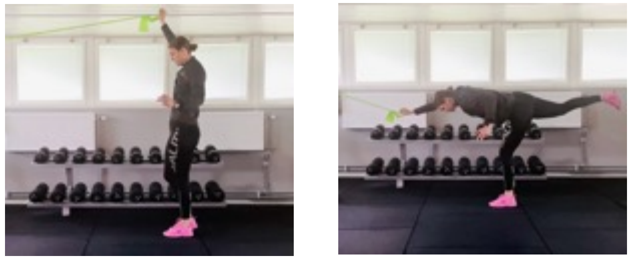

Level C **One arm diver in overhead position with an elastic band** **2-3x30 seconds**

Instructions: *Same as level A but holding an elastic band that is fixated in front of the body.*

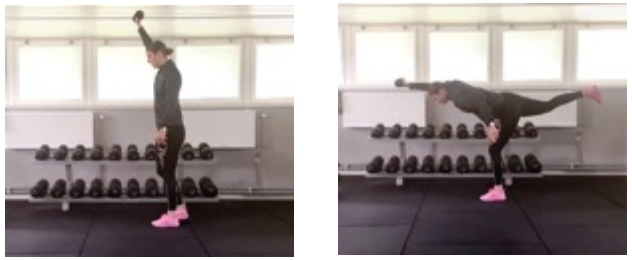

Level D **One arm diver in overhead position with a dumbbell** **2-3x30 seconds**

Instructions: *Same as level A but holding a dumbbell*

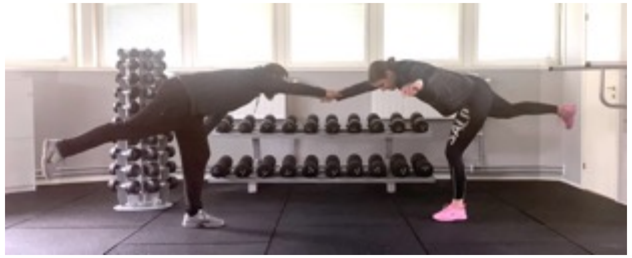

Partner exercise **One arm in diver overhead position arm wrestling** **2-3x15 seconds**

Instructions: *Same as level A but the partner pushing the arm downward*

5. Trunk rotational strength

Level A | **Resisted trunk rotations with elastic band** | **2-3x30 seconds**

Instructions: *Stand shoulder wide with an elastic band fixated the side of the body. Hold the arms straight in front of the chest. Rotated the upper body as far as possible while maintaining the body in a straight line.*

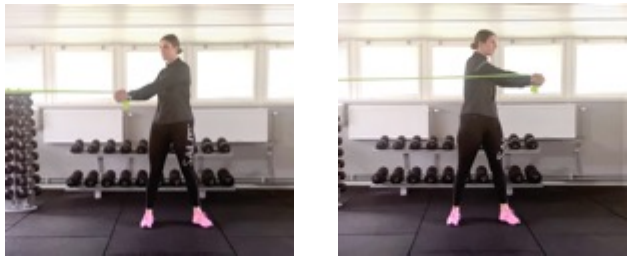

Level B | **Diagonal pattern in lunge position with elastic band** | **2-3x30 seconds**

Instructions: *Stand in a lunge position with an elastic band fixated the behind the body. Hold the elastic band in an overhead position and rotate the upper body so the hands ends up at the front knee.*

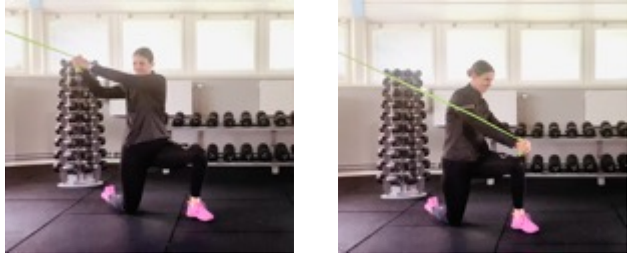

Level C | **Trunk rotation in throwing position with elastic band** | **2-3x30 seconds**

Instructions: *Stand shoulder wide with an elastic band fixated the behind the body. Hold the arm in an overhead position. Rotate the upper body while maintaining the body in a straight line and the arm in the overhead position.*

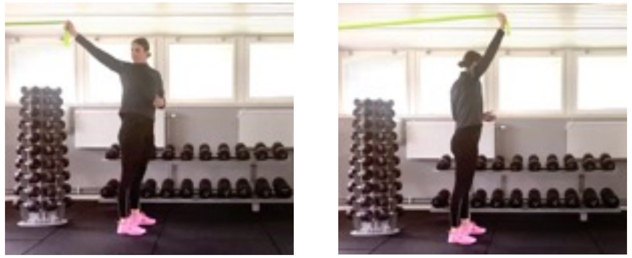

Level D | **Resisted throwing motion with elastic band** | **2-3x30 seconds**

Instructions: *Stand with one foot in front of the other with an elastic band fixated the behind the body. Hold the arm in a throwing position and perform a throwing motion and go back to the starting position again.*

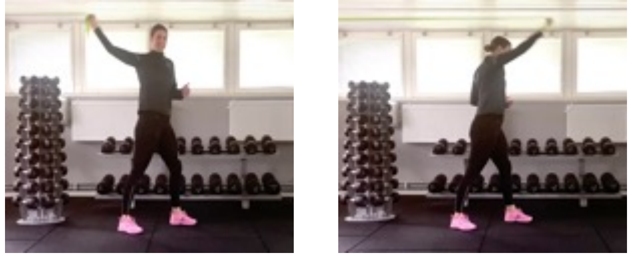

Partner exercise | **Isometric trunk rotation** | **2-3x15 seconds**

Instructions: *Maintain the body and arms straight while the partner pushes the arms from side to side.*

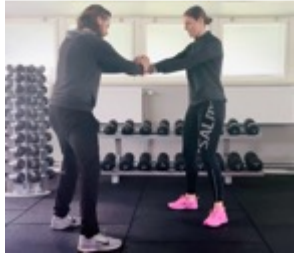

6. Handball throwing programme

The handball throwing programme is recommended to be performed every second day during the off-season (June-August). Start at level A and progress to the next level after one week until you reach level D.

Level A **2 sets of 15 throws at 50% of maximum velocity and 10 throws at 70 % of maximum velocity**

Level B **2 sets of 15 throws at 50% of Maximum velocity, 10 throws at 70% of maximum velocity and 5 throws at 90% of maximum velocity**

Level C **2 sets of 15 throws at 60% of maximum velocity, 10 throws at 80% of maximum velocity and 5 throws at 100% of maximum velocity**

Level D **2 sets of 15 throws at 70% of maximum velocity , 10 throws at 90% of maximum velocity and 10 throws at 100% of maximum velocity**
