# Supplementary material for: The Effect of Shoulder and Knee Exercise Programmes on the Risk of Shoulder and Knee Injuries in Adolescent Elite Handball Players: A Three-Armed Cluster Randomised Controlled Trial
Source: Sports Med Open. 2022 Jul 14;8:91. doi: 10.1186/s40798-022-00478-z (PMC9283550; doi:10.1186/s40798-022-00478-z)

Supplementary file 2. Knee Control exercise programme

The effect of shoulder and knee exercise programmes on the risk of shoulder and knee injuries in adolescent elite handball players: a three-armed cluster randomised controlled trial.

Martin Asker [1,2,3], Martin Hägglund [4,5], Markus Waldén [4,6,7], Henrik Källberg [1,8], Eva Skillgate [1,2]

[1] Handball Research Group, Musculoskeletal & Sports Injury Epidemiology Center, Department of health promotion science, Sophiahemmet University, Stockholm, Sweden

[2] Unit for Intervention and Implementation Research in worker health, Institute of Environmental Medicine, Karolinska Institutet, Solna, Sweden

[3] Naprapathögskolan, Scandinavian College of Naprapathic Manual Medicine, Stockholm, Sweden

[4] Sport Without Injury ProgrammE (SWIPE), Linköping University, Linköping, Sweden

[5] Unit of Physiotherapy, Department of Health, Medicine and Caring Sciences, Linköping University, Linköping, Sweden

[6] Unit of Community Medicine, Department of Health, Medicine and Caring Sciences, Linköping University, Linköping, Sweden

[7] Ortho & Spine Center Skåne, Malmö, Sweden

[8] Unit of analysis, Department of Public Health, Analysis and Data Management, Public Health Agency of Sweden, Stockholm, Sweden

Corresponding author:
Martin Asker
martin.asker@shh.se

Supplementary file 2. Knee Control exercise programme.

KNEE CONTROL PROGRAMME

Knee Control (Knäkontroll, SISU Idrottsböcker, Sweden 2005) is an injury prevention exercise programme that focuses on lower limb and trunk strength, neuromuscular control, balance and jumping/landing technique. For this trial minor modifications were made to fit a handball setting.

Choose one exercise from each category 1-5 at a difficulty level where you can perform the exercise with good technique but it is still challenging. During the off-season and pre-season (June to August) the programme is performed 3 times per week with 3 sets of each exercise, with a pace of 1-2 second from starting position to end position.

During the handball season (September to May) the programme is meant to be performed in conjunction with the normal handball warm-up. During the handball season the programme is performed in 2 sets of each exercise, with a pace of 1-2 second from starting position to end-position.

1. One legged knee squats

Level A — **One legged knee squat without a ball** — **2-3x30 seconds**

Instructions: *Hands on hip. Perform a single leg squat with a. smooth motion and try to maintain good knee control with the knee stable* and aligned with the foot.

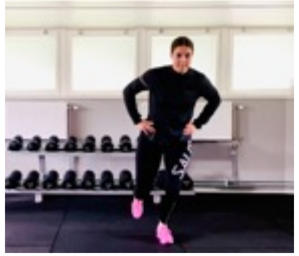

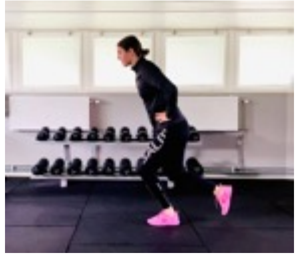

Level B — **One legged knee squat with a ball above the head** — **2-3x30 seconds**

Instructions: *Same as Level A but with the ball above the head*

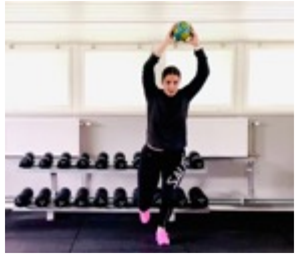

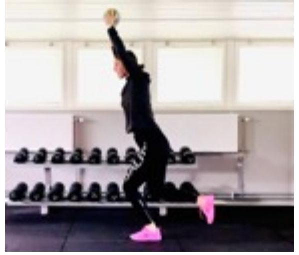

Level C — **One legged knee squat with trunk rotation** — **2-3x30 seconds**

Instructions: *Same as Level A but with the ball in front and in the end position, a trunk rotation is performed*

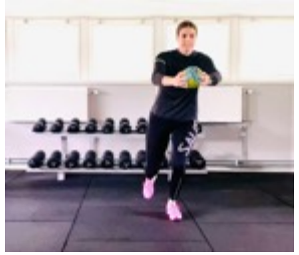

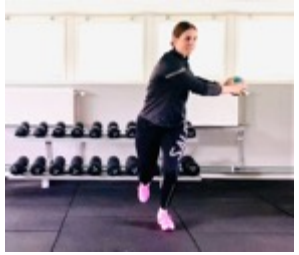

Level D — **One legged knee squat with passing the ball** — **2.-3x30 seconds**

Instructions: *Same as Level A and passing the ball during the exercise*

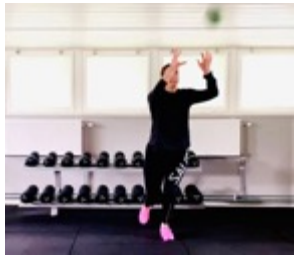

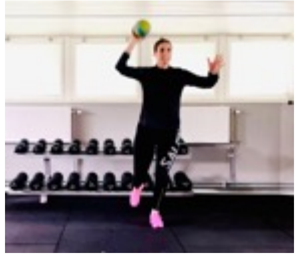

Partner exercise — **Single leg arm wrestling** — **2-3x15 seconds**

Instructions: *Position; One legged stance with a slightly bent knee Try to push the partner out of balance.*

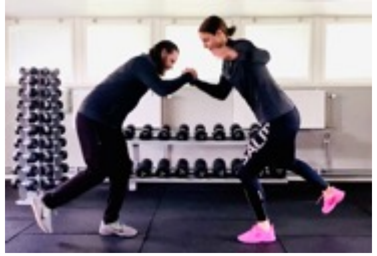

2. Pelvic lifts

Level A — **Pelvic lifts on two feet** — **2-3x30 seconds**

Instructions: *Supine position. Both feet on the ground. Hands across the chest. Lift the pelvis up to a neutral position while maintaining the back straight*

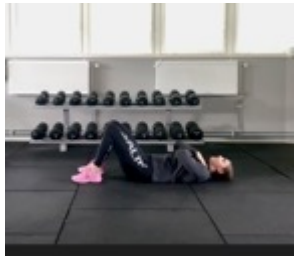
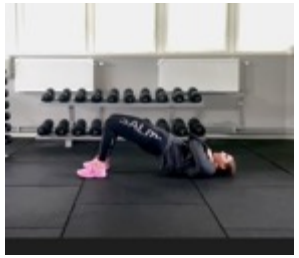

Level B — **Pelvic lift on one foot** — **2-3x30 seconds**

Instructions: *Same as level A but one foot on the ground*

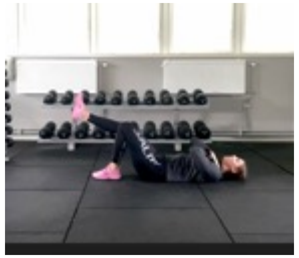
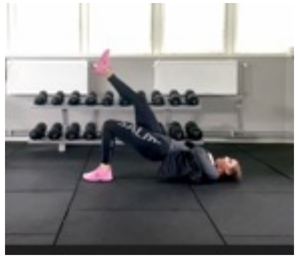

Level C — **Pelvic lift on one foot with a ball** — **2-3x30 seconds**

Instructions: *Same as level B but with a ball squeezed between the knees*

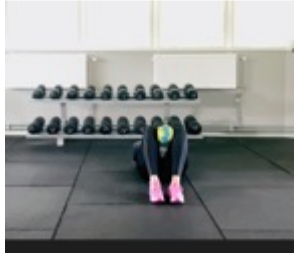
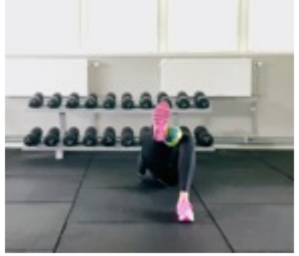

Level D — **Pelvic lift on one foot – foot on bench/ball** — **2-3x15-30 seconds**

Instructions: *Same as level B but foot on a bench or the ball*

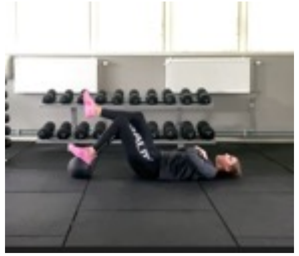
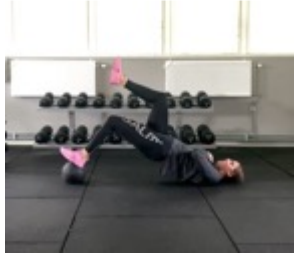

Partner exercise — **Side plank pushes** — **2-3x15-30 seconds**

Instructions: *Side plank position. Maintain the body in a straight line. Try to push the partner out of balance.*

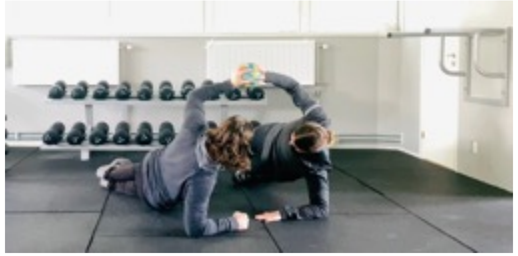

3. Two legged knee squats

Level A — **Two legged knee squats without a ball** — **2-3x30 seconds**

Instructions: *Shoulder-wide standing position with hands on the hips, a straight back and trunk upright. Perform a smooth squat as deep a possible without lifting your heels from the ground.*

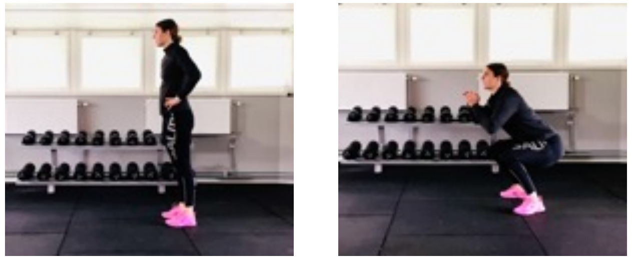

Level B — **Two legged knee squats with ball in front** — **2-3x30 seconds**

Instructions: *Same as Level A but with the ball in front*

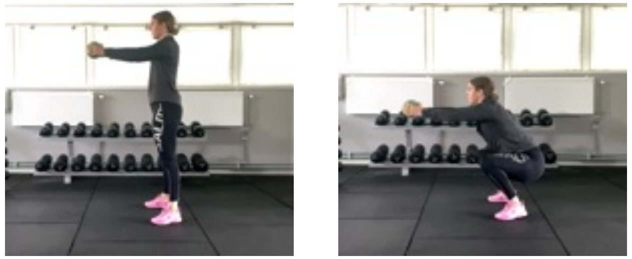

Level C — **Two legged knee squats with ball above the head** — **2-3x15-30 seconds**

Instructions: *Same as Level A but with the ball above the head*

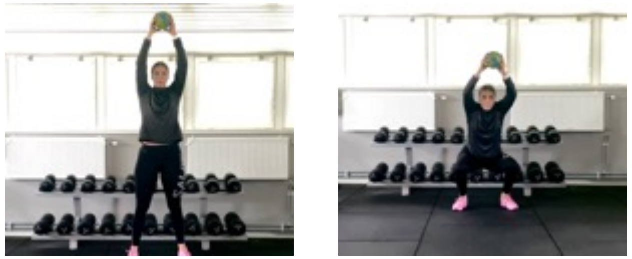

Level D — **Two legged knee squats with ball above the head – up on toe** — **2-3x15-30 seconds**

Instructions: *Same as Level C but in the end position go up on your toes*

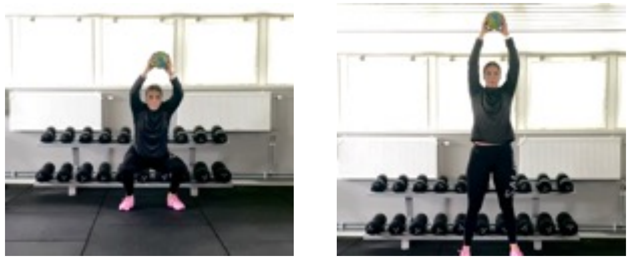

Partner exercise — **Two legged knee squats – Passing your partner** — **2-3x15-30 seconds**

Instructions: *Same as Level A but passing the ball to your partner*

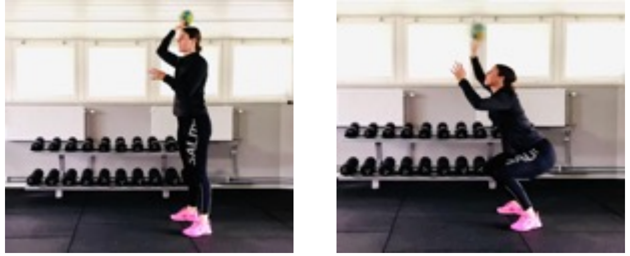

4. The bench

Level A **Bench – On knees and forearms** **2-3x30 seconds**

Instructions: *Elbows are placed beneath the shoulder.*
Lift the body and maintain it in a straight line

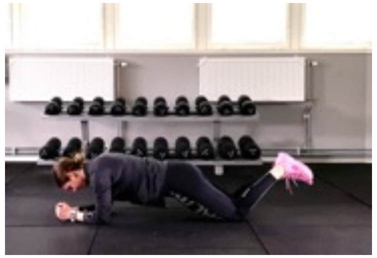

Level B **Bench – On knees with arm raises** **2-3x30 seconds**

Instructions: *Same as Level A but raise one arm at a time straight forward*

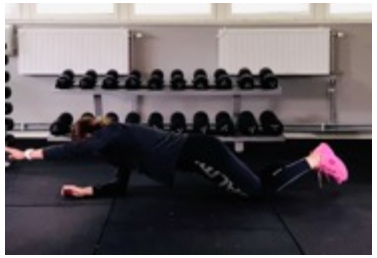

Level C **Bench – On toes and forearms** **2-3x30 seconds**

Instructions: *Same as Level A but support on toes instead of knees*

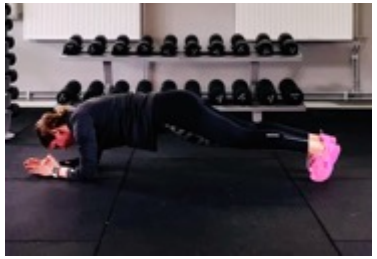

Level D **Bench - On toes with leg-lifts** **2-3x30 seconds**

Instructions: *Same as Level C but raise one leg at a time*

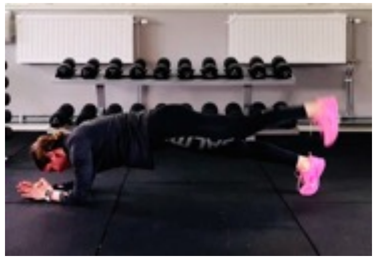

Partner exercise **Bench – Hit the partner’s hands** **2-3x15-30 seconds**

Instructions: *In a push-up position try to hit your partner´s hands*

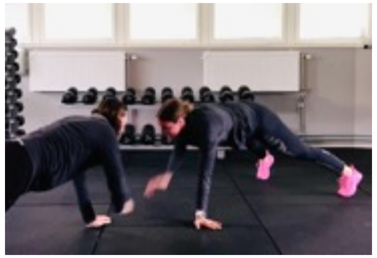

5. Lunges

Level A — **Lunges without a ball** — **2-3x30 seconds**

Instructions: *Stand with hand on the hips. Take a deep step forward so the forward knee is flexed 90°. The motion should be smooth and try to maintain a good knee control with the the knee aligned with the foot*

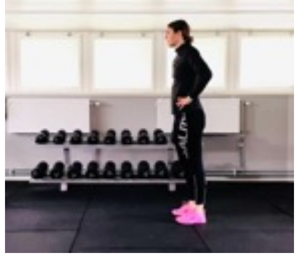
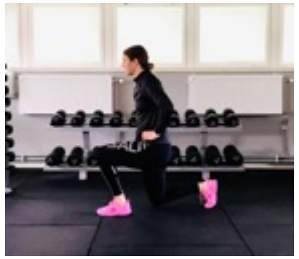

Level B — **Lunges with a ball above the head** — **2-3x30 seconds**

Instructions: *Same as Level A but with the ball above the head*

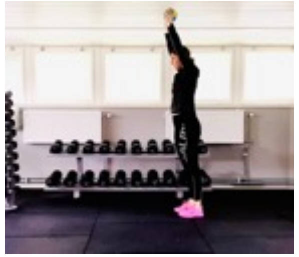
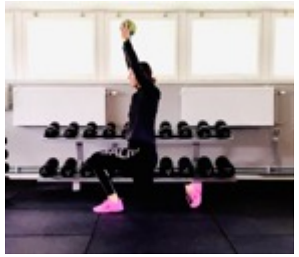

Level C — **Diagonal lunges with a ball in front** — **2-3x30 seconds**

Instructions: *Same as Level A but with the ball in front and*

the step is taken diagonally

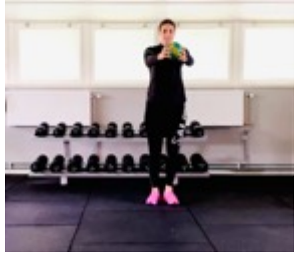
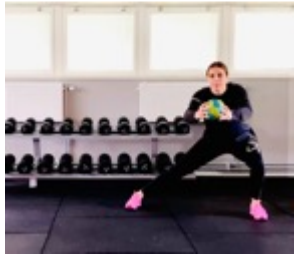

Level D — **Lunges with a ball in front with trunk rotation** — **2-3x30 seconds**

Instructions: *Same as Level A but with the ball in front and in the end position, a trunk rotation is performed*

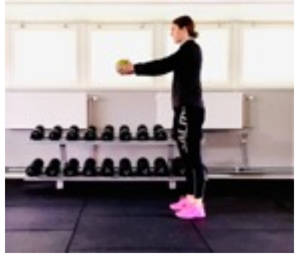
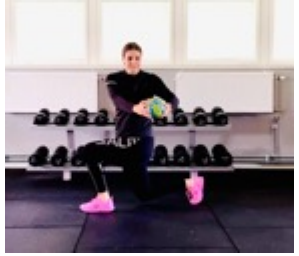

Partner exercise — **Lunges – Passing your partner** — **2-3x15 seconds**

Instructions: *Same as Level A but passing the ball to your partner*

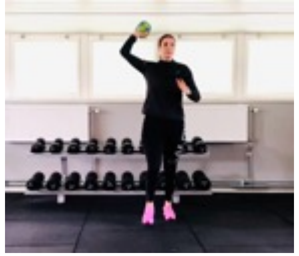
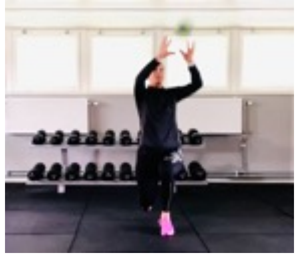

6. Jumping/landing

Level A — **Forward jumps on one leg** — **2-3x30 seconds**

Instructions: *Stand on one leg and make a long forward jump and land on the same foot with a soft landing and "freeze" this position. Try to maintain good knee control*

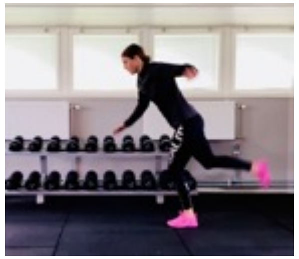
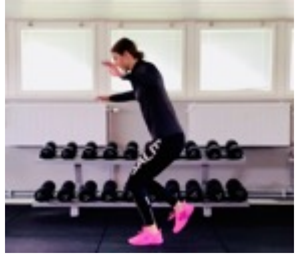

Level B — **Jumping on the spot with 90° rotation** — **2-3x30 seconds**

Instructions: *Stand on one leg and jump on the spot. In the air* turn 90° and land with a soft landing and "freeze" *the position shortly before performing another jump with a 90° turn.*

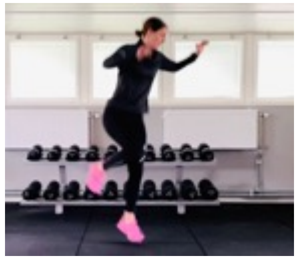
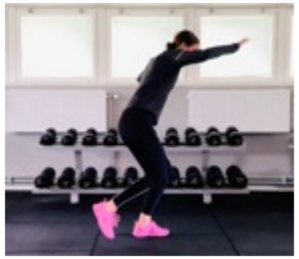

Level C — **Side jumps** — **2-3x30 seconds**

Instructions: *Stand on one leg and make long side-jump and land on the opposite foot with a soft landing and "freeze" this position. Try to maintain good knee control*

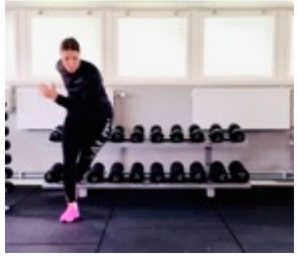
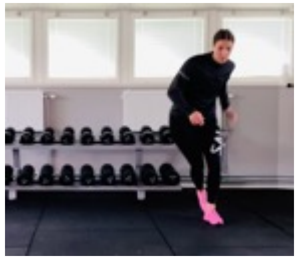

Level D — **Drop jumps – two leg/one leg** — **2-3x30 seconds**

Instructions: *Stand on a board and drop down and land on one foot, and instantly perform a jump on the same foot and make a soft landing and "freeze" that position. Try to maintain good knee control. Can also be performed on two feet*

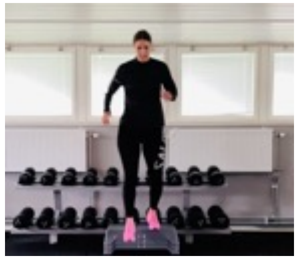
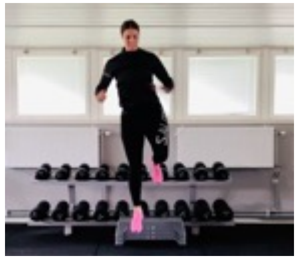

Partner exercise — **Drop jumps – two leg/one leg with passes** — **2-3x30 seconds**

Instructions: *Same as Level D but passing the ball to your partner*

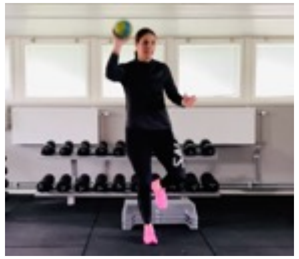
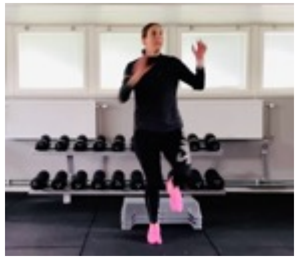

Supplement: Supplementary file 2 — Additional file 2: Knee Control exercise programme. [file 40798_2022_478_MOESM2_ESM.pdf]
