# Supplementary material for: The Effect of Shoulder and Knee Exercise Programmes on the Risk of Shoulder and Knee Injuries in Adolescent Elite Handball Players: A Three-Armed Cluster Randomised Controlled Trial
Source: Sports Med Open. 2022 Jul 14;8:91. doi: 10.1186/s40798-022-00478-z (PMC9283550; doi:10.1186/s40798-022-00478-z)
Supplement: Supplementary file 3 — Additional file 3: Acute injury report card. [file 40798_2022_478_MOESM3_ESM.docx]

**Supplementary file 3. Acute injury report form**

**The effect of shoulder and knee exercise programmes on the risk of shoulder and knee injuries in adolescent elite handball players: a three-armed cluster randomised controlled trial**

Martin Asker ^1,2,3^, Martin Hägglund ^4,5^, Markus Waldén ^4,6,7^, Henrik Källberg ^1,8^, Eva Skillgate ^1,2^

^1^ Handball Research Group, Musculoskeletal & Sports Injury Epidemiology Center, Department of health promotion science, Sophiahemmet University, Stockholm, Sweden

^2^ Unit for Intervention and Implementation Research in worker health, Institute of Environmental Medicine, Karolinska Institutet, Solna, Sweden

^3^ Naprapathögskolan, Scandinavian College of Naprapathic Manual Medicine, Stockholm, Sweden

^4^ Sport Without Injury ProgrammE (SWIPE), Linköping University, Linköping, Sweden

^5^ Unit of Physiotherapy, Department of Health, Medicine and Caring Sciences, Linköping University, Linköping, Sweden

^6^ Unit of Community Medicine, Department of Health, Medicine and Caring Sciences, Linköping University, Linköping, Sweden

^7^ GHP Ortho & Spine Center Skåne, Malmö, Sweden

^8^ Unit of analysis, Department of Public Health, Analysis and Data Management, Public Health Agency of Sweden, Stockholm, Sweden

Corresponding author:

Martin Asker

martin.asker@shh.se

**Supplementary file 3. Acute injury report form**

**ID number:** _________________________ **School:** _____________________________________________

**Date of injury:** ______________________ **Date of return to full participation:** ______________________

**Injured body part:**

Head / face Shoulder / clavicula Hip / groin

Neck / cervical spine Upper arm Thigh

Sternum / ribs / upper back Elbow Knee

Abdomen Forearm Lower leg / Achilles tendon

Low back / sacrum /pelvis Wrist Ankle

Hand / finger / thumb Foot / toe

**Injured side of the body**

Right Left Bilateral / central

**Type of injury**

Concussion Meniscus / cartilage injury Hematoma / contusion

Fracture Muscle rupture / strain / tear / cramp Abrasion

Other bone injury Tendon injury / tendinopathy / bursitis Laceration

Dislocation / subluxation Synovitis / joint swelling Nerve injury

Sprain / ligament injury Overuse injury unspecified Dental injury

Other injury (specify): _______________________________________________________________________

**Diagnosis:** __________________________________________________________________________________

**Was it a re-injury?**

No Yes (specify the date of return to sport from the previous injury): ______________________

**Was the injury caused by overuse (gradual onset) or trauma (acute onset)?**

Overuse Trauma

**Injury mechanism**

Running / sprinting Jumping / Landing Hit by the ball Kicked by another player

Twist /turn Fall Collision with another player Hit by another player

Shot Stretching Collision with equipment Blocking

Pass Gliding / slipping Tackling (defense) Use of arm / elbow (wrestling)

Sidestep Overuse Tackling (attack)

Other acute mechanism (describe): ___________________________________________________________________

**Other comments:** ____________________________________________________________________________

**Report filled in by (name):** ___________________________ **Report date:** _____________________________
