# Supplementary material for: The Effect of Shoulder and Knee Exercise Programmes on the Risk of Shoulder and Knee Injuries in Adolescent Elite Handball Players: A Three-Armed Cluster Randomised Controlled Trial
Source: Sports Med Open. 2022 Jul 14;8:91. doi: 10.1186/s40798-022-00478-z (PMC9283550; doi:10.1186/s40798-022-00478-z)
Supplement: Supplementary file 4 — Additional file 4: Handball match and training time and number of shoulder and knee injuries and problems during the follow-up. [file 40798_2022_478_MOESM4_ESM.docx]

**Supplementary file 4.** **Handball match and training time and number of new shoulder and knee injuries and problems during the follow-up**

**The effect of shoulder and knee exercise programmes on the risk of shoulder and knee injuries in adolescent elite handball players: a three-armed cluster randomised controlled trial**

Martin Asker ^1,2,3^, Martin Hägglund ^4,5^, Markus Waldén ^4,6,7^, Henrik Källberg ^1,8^, Eva Skillgate ^1,2^

^1^ Handball Research Group, Musculoskeletal & Sports Injury Epidemiology Center, Department of health promotion science, Sophiahemmet University, Stockholm, Sweden

^2^ Unit for Intervention and Implementation Research in worker health, Institute of Environmental Medicine, Karolinska Institutet, Solna, Sweden

^3^ Naprapathögskolan, Scandinavian College of Naprapathic Manual Medicine, Stockholm, Sweden

^4^ Sport Without Injury ProgrammE (SWIPE), Linköping University, Linköping, Sweden

^5^ Unit of Physiotherapy, Department of Health, Medicine and Caring Sciences, Linköping University, Linköping, Sweden

^6^ Unit of Community Medicine, Department of Health, Medicine and Caring Sciences, Linköping University, Linköping, Sweden

^7^ GHP Ortho & Spine Center Skåne, Malmö, Sweden

^8^ Unit of analysis, Department of Public Health, Analysis and Data Management, Public Health Agency of Sweden, Stockholm, Sweden

Corresponding author:

Martin Asker

martin.asker@shh.se

**Supplementary file 4. Handball match and training time and number of new shoulder and knee
injuries and problems during the follow-up**

|  | **Shoulder Group** | | **Knee Group** | | **Control Group** | |
| --- | --- | --- | --- | --- | --- | --- |
|  | **Males**  **(n=118)** | **Females**  **(n=81)** | **Males**  **(n=110)** | **Females**  **(n=106)** | **Males**  **(n=111)** | **Females**  **(n=101)** |
| Mean handball match exposure, h/week (SD) | 0.5 (0.8) | 0.5 (0.8) | 0.5 (0.8) | 0.4 (0.8) | 0.5 (0.8) | 0.5 (0.9) |
| Mean handball training exposure, h/week (SD) | 3.6 (3.1) | 3.3 (3.1) | 3.6 (3.0) | 2.9 (2.6) | 3.8 (3.3) | 3.4 (3.1) |
| Mean strength & conditioning exposure, h/week (SD) | 4.3 (2.8) | 3.6 (2.5) | 3.3 (2.3) | 3.1 (2.1) | 3.9 (2.6) | 3.5 (2.4) |
| Shoulder injury,  n (n with acute/gradual onset) ^*^ | 11 (2/9) | 10 (1/9) | 14 (3/11) | 19 (3/16) | 24 (4/20) | 22 (4/18) |
| Time-loss shoulder injury,  n (n with acute/gradual onset) ^†^ | 11 (3/8) | 7 (1/6) | 15 (4/11) | 17 (6/11) | 25 (4/21) | 17 (6/11) |
| Substantial shoulder problem,  n (n with acute/gradual onset) ^‡^ | 13 (3/10) | 15 (2/13) | 17 (4/13) | 28 (3/25) | 26 (4/22) | 26 (2/24) |
| Any shoulder problem,  n (n with acute/gradual onset) ^§^ | 26 (3/16) | 26 (2/24) | 37 (5/32) | 40 (4/36) | 42 (5/37) | 46 (4/42) |
| Knee injury, n  (n with acute/gradual onset) ^*^ | 28 (5/23) | 25 (4/21) | 24 (6/18) | 20 (7/13) | 25 (5/20) | 34 (13/21) |
| Time-loss knee injury, n  (n with acute/gradual onset) ^†^ | 30 (5/25) | 22 (8/14) | 22 (7/15) | 22 (7/15) | 27 (8/19) | 36 (15/21) |
| Substantial knee problem, n  (n with acute/gradual onset) ^‡^ | 31 (5/26) | 27 (4/23) | 25 (6/19) | 20 (5/15) | 30 (4/26) | 37 (13/24) |
| Any knee problem, n  (n with acute/gradual onset) ^§^ | 46 (4/42) | 31 (4/27) | 46 (6/40) | 47 (6/41) | 51 (7/44) | 57 (14/43) |

SD – standard deviation
^*^ Reporting a score of 40 points or more for shoulder or knee problems, respectively with the Oslo Sports Trauma Research Center Overuse Injury Questionnaire (OSTRC-O)

^†^ Reporting at least a reduced participation or inability to participate due to a shoulder or knee problems, respectively with the OSTRC-O

^‡^ Reporting at least a moderate reduction in training volume or performance due to a shoulder or knee problems, respectively with the OSTRC-O
^§^ Reporting anything but “full participation without any shoulder/knee problems”, respectively with the OSTRC-O
