# Supplementary material for: The Effect of Shoulder and Knee Exercise Programmes on the Risk of Shoulder and Knee Injuries in Adolescent Elite Handball Players: A Three-Armed Cluster Randomised Controlled Trial
Source: Sports Med Open. 2022 Jul 14;8:91. doi: 10.1186/s40798-022-00478-z (PMC9283550; doi:10.1186/s40798-022-00478-z)
Supplement: Supplementary file 5 — Additional file 5: Weekly prevalence of shoulder and knee problems. [file 40798_2022_478_MOESM5_ESM.docx]

**Supplementary file 5. Weekly prevalence of any and substantial shoulder and knee problems**

**The effect of shoulder and knee exercise programmes on the risk of shoulder and knee injuries in adolescent elite handball players: a three-armed cluster randomised controlled trial**

Martin Asker ^1,2,3^, Martin Hägglund ^4,5^, Markus Waldén ^4,6,7^, Henrik Källberg ^1,8^, Eva Skillgate ^1,2^

^1^ Handball Research Group, Musculoskeletal & Sports Injury Epidemiology Center, Department of health promotion science, Sophiahemmet University, Stockholm, Sweden

^2^ Unit for Intervention and Implementation Research in worker health, Institute of Environmental Medicine, Karolinska Institutet, Solna, Sweden

^3^ Naprapathögskolan, Scandinavian College of Naprapathic Manual Medicine, Stockholm, Sweden

^4^ Sport Without Injury ProgrammE (SWIPE), Linköping University, Linköping, Sweden

^5^ Unit of Physiotherapy, Department of Health, Medicine and Caring Sciences, Linköping University, Linköping, Sweden

^6^ Unit of Community Medicine, Department of Health, Medicine and Caring Sciences, Linköping University, Linköping, Sweden

^7^ GHP Ortho & Spine Center Skåne, Malmö, Sweden

^8^ Unit of analysis, Department of Public Health, Analysis and Data Management, Public Health Agency of Sweden, Stockholm, Sweden

Corresponding author:

Martin Asker

martin.asker@shh.se

**Supplementary file 5. Weekly prevalence of any and substantial shoulder and knee problems**

The weekly prevalence of any and substantial problems is defined as the number of players who reported any and substantial shoulder or knee problems, with the OSTRC-O respectively in each group, divided by the total number of player reports in the group for each week.

Weekly prevalence of *any shoulder problems*


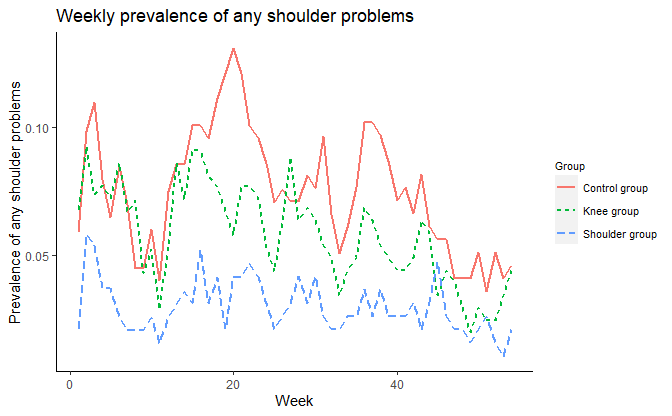


Prevalence of any shoulder problems

Weekly prevalence of *substantial shoulder problems*


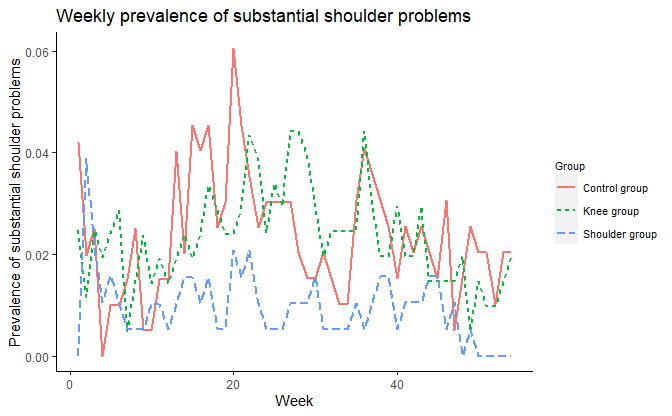


Prevalence of substantial shoulder problems

Weekly prevalence of *any knee problems*


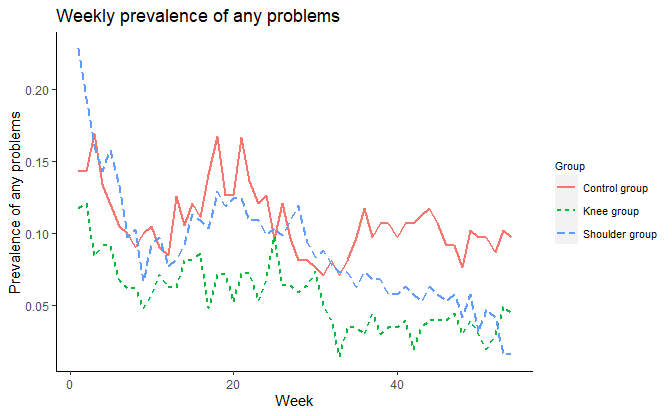


Prevalence of any knee problems

Weekly prevalence of *substantial knee problems*


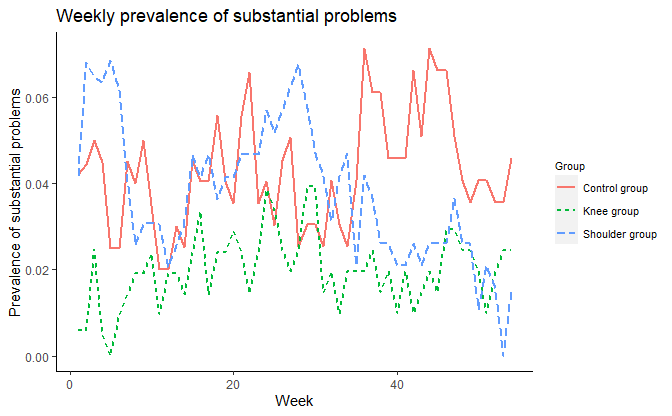


Prevalence of substantial knee problems
